# Effect of larger corneal spherical aberration in improving the near visual acuity of eyes implanted with the TECNIS Symfony

**DOI:** 10.3389/fmed.2023.1094966

**Published:** 2023-02-23

**Authors:** Dandan Wang, Chunlu Liu, Weichen Guan, Ziyi Lu, Yinying Zhao, Yune Zhao

**Affiliations:** ^1^Eye Hospital and School of Ophthalmology and Optometry, Wenzhou Medical University, Wenzhou, Zhejiang, China; ^2^National Clinical Research Center for Ocular Disease, Eye Hospital, Wenzhou Medical University, Wenzhou, Zhejiang, China; ^3^Zhejiang University School of Medicine Children’s Hospital, Hangzhou, Zhejiang, China; ^4^Joint Shantou International Eye Center (JSIEC), The Shantou University, The Chinese University of Hong Kong, Shantou, Guangdong, China

**Keywords:** corneal spherical aberration, ocular spherical aberration, visual quality, near vision quality, contrast sensitivities

## Abstract

**Purpose:**

To explore the effect of corneal spherical aberration on the visual acuity and visual quality of eyes implanted with the TECNIS Symfony intraocular lens (IOL).

**Methods:**

A total of 43 patients with age-related cataract (60 eyes) undergoing phacoemulsification and TECNIS Symfony IOL implantation were enrolled in this study. The uncorrected distance (UDVA), intermediate (UIVA), near visual acuity (UNVA), corrected distance visual acuity (CDVA), contrast sensitivity, and ocular spherical aberration were recorded 3 months after surgery. Preoperative and postoperative corneal spherical aberration were also measured using the iTrace device. Objective scattering index (OSI), modulation transfer function cut-off frequency (MTF cut-off), and Strehl ratio (SR) were measured by the Optical Quality Analyzing System. Catquest-9SF questionnaire were applied too. Spearman’s correlation analysis was used to evaluate the relationship between spherical aberration and visual quality parameters.

**Results:**

Patients were satisfied with their postoperatively visual quality. And the postoperative logMAR UDVA, UIVA, UNVA, and CDVA was 0.05 ± 0.07, 0.04 ± 0.06, 0.15 ± 0.07, and 0.03 ± 0.05, respectively. The mean preoperative corneal spherical aberration was 0.24 ± 0.10 μm, which is the only factor influencing postoperatively UNVA, and it was negatively correlated with UNVA and glare contrast sensitivity under 18 cpd (cycle/degree, cpd) spatial frequency (*r* = −0.403, −0.300, −0.360; all *P* < 0.05). Additionally, the greater the residual spherical aberration of the cornea, the better the near vision after operation. The mean postoperative ocular spherical aberration was −0.03 ± 0.07 μm, it was not correlated with visual acuity, contrast sensitivity, and visual quality (all *P* > 0.05).

**Conclusion:**

Preoperative positive spherical aberration can benefit near vision while decrease contrast sensitivities at high spatial frequencies when implanted with the TECNIS Symfony IOL.

## Introduction

With the advent of refractive cataract surgery, ophthalmologists and patients have higher expectations of visual quality after cataract surgery. Multifocal aspherical intraocular lenses (IOLs) have greatly decreased spectacle dependence and created to compensate for the spherical aberration of the cornea and to lessen total ocular spherical aberration in pseudophakic eyes. However, even after the monofocal aspherical IOLs implantation, the optimal value of target ocular spherical aberration remains controversial. Previous studies have revealed that implantation with aspherical IOLs can improve the visual quality (1, 2). Denoyer et al. (3) reported that bilateral implantation of an IOL with no aberration resulted in better quality of near vision. Other researchers though that completely correcting spherical aberration will damage depth of field and near acuity (4–6). Rocha et al. (5) found that the reduction of total spherical aberration after aspheric IOL implantation may degrade distance-corrected near and intermediate visual acuity. Nochez et al. (7) reported that some residual positive spherical aberrations (0. 07–0.10 μm) can increase the depth of focus and improve the near visual acuity in eyes implanted with aspherical monofocal IOLs.

TECNIS Symfony IOL (Johnson & Johnson Vision, Santa Ana, CA, USA) is a single-piece, hydrophobic acrylic extended depth-of-focus (EDOF) IOL with an asphericity of −0.27 μm (8). As a hybrid EDOF IOL, it provides excellent far, intermediate visual acuity and good visual quality. But the near visual acuity is not always good enough (9, 10). Interestingly, we found that in the clinic some patients had good near vision, while some had poor near vision despite having the same TECNIS Symfony IOL implantation. Does the spherical aberration play a role in improving near vision? In addition, how does the spherical aberration affect the visual quality after surgery?

Therefore, this study aimed to explore the influence of spherical aberration on the visual acuity and visual quality, especially the near vision, after TECNIS Symfony IOL implantation.

## Patients and methods

### Patients

This prospective study was conducted at the Eye Hospital of Wenzhou Medical University from January 2019 to October 2021. Sixty eyes of 43 age-matched cataract patients who underwent uneventful phacoemulsification and TECNIS Symfony IOL implantation were enrolled. All surgeries were performed by the same surgeon (Z.Y.E.) using topical anesthesia. Patients with other ocular diseases (such as keratopathy, glaucoma, uveitis, and fundus disease), history of intraocular or corneal surgery, and any complications intra- and post-operative were excluded. All procedures were conducted following the tenets of the Declaration of Helsinki, and the study design was approved by the Institutional Ethics Committee of Wenzhou Medical University, Zhejiang Province, China. All study participants provided informed consent.

### Examinations and measurements

All patients underwent a comprehensive ophthalmological examination. The preoperative examination data included uncorrected distance visual acuity (UDVA), corrected distance visual acuity (CDVA), intraocular pressure, axial length, and corneal astigmatism measured by the IOL Master 700 (Carl Zeiss, Jena, Germany) and corneal spherical aberration measured by the iTrace aberrometer (Tracey Technologies, Houston, TX, USA) in a 6-mm range. The Barrett Universal II formula was used to determine the IOL power, and the postoperative target diopter was set to mild myopia (0–0.5D).

Postoperative examinations were conducted 3 months after cataract surgery. The data included UDVA, CDVA, uncorrected intermediate visual acuity (UIVA) at a distance of 80 cm, uncorrected near visual acuity (UNVA) at a distance of 40 cm, using Snellen visual charts and then converted into logarithm of the minimum angle of resolution (logMAR) notation. Astigmatism, and postoperative ocular spherical aberration, coma, trefoil in a 4-mm range was also recorded. After correcting refractive errors, CSV-1000HGT (Vector Vision, Dayton, OH, USA) was applied to measure the contrast sensitivity (CS) with and without glare after adapting the patient to scotopic conditions. Spatial frequencies of 3, 6, 12, and 18 cpd (cycle/degree, cpd) were used, which were then converted into base 10 logarithmic units for statistical analysis. Objective visual quality parameters, that is, the objective scatter index (OSI), modulation transfer function (MTF), and Strehl ratio (SR) using an optical quality analysis system (OQAS, Visiometrics SL, Terrassa, Spain) were recorded. Subjective visual quality was evaluated using the Catquest-9SF questionnaire with four response options for perceived difficulty in vision (4 = very great difficulty; 3 = great difficulty; 2 = some difficulty; 1 = no difficulty), and the Quality of Vision (QoV) questionnaire wherein the patients rated 10 visual symptoms with four response levels (0, 1, 2, 3; higher scores indicated worse photic phenomena).

### Statistical analyses

Statistical analyses were performed using Statistical Package for the Social Sciences (SPSS) (version 22.0; SPSS, Inc., Chicago, IL, USA). The normality of the evaluation data was tested by the Shapiro–Wilk test. If the data followed a normal distribution, parametric analysis was performed, and if not, non-parametric statistical analysis was used. Categorical data, such as halos and glares, were expressed as frequencies with percentages (n%). Spearman’s correlation was used to evaluate the influence of preoperative corneal spherical aberration and postoperative ocular spherical aberration on the visual quality and visual acuity of eyes implanted with TECNIS Symfony IOL, and factors with *P* < 0.2 were included in the further multiple linear regression. The multiple linear regression was used to analyze the impact of eye parameters on postoperative UNVA. Statistical significance was set at *P* < 0.05. The sample size calculation suggested that a sample of 55 would achieve a power of 80% and a level of significance of 5% for the detection of a significant correlation between preoperative corneal spherical aberration and postoperative UNVA.

## Results

Sixty eyes from 43 patients (16 men and 27 women) were enrolled. Of these, 17 eyes had bilateral cataracts, and 26 had unilateral cataracts. The mean patient age was 66 ± 10 years. Before the operation, the mean corneal spherical aberrations of 6-mm measurement were 0.24 ± 0.10 μm (ranging from 0.07 to 0.47 μm), the mean logarithmic values of CDVA and UDVA were 0.35 ± 0.44 and 0.54 ± 0.40, respectively. The axial length and corneal astigmatism were 23.56 ± 0.99 mm and −0.47 ± 0.30 diopter, respectively. After the operation, the ocular spherical aberrations of 4-mm measurement were −0.03 ± 0.07 μm (ranging from −0.23 to 0.14 μm) (Table 1). There were no intraoperative or postoperative complications. The capsule was transparent at the end of the follow-up.

**TABLE 1:** Pre- and post-operative data of patients.

	Pre-operative	Post-operative
Age (year)	66 ± 10 (37, 85)	–
Axial length (mm)	23.56 ± 0.99 (21.92, 25.89)	–
Corneal astigmatism (D)	−0.47 ± 0.30 (−1.54, 0)	–
Corneal spherical aberration (6 mm)	0.24 ± 0.10 (0.07, 0.47)	–
Ocular spherical aberration (4 mm)	–	−0.03 ± 0.07 (−0.23, 0.14)
UDVA (log MAR)	0.54 ± 0.40 (0.10, 2.30)	0.05 ± 0.07 (−0.1, 0.2)
CDVA (log MAR)	0.35 ± 0.44 (0, 2.30)	0.03 ± 0.05 (0, 0.2)
UIVA (log MAR)	–	0.04 ± 0.06 (−0.10, 0.2)
UNVA (log MAR)	–	0.15 ± 0.07 (0, 0.3)

UDVA, uncorrected distance visual acuity; CDVA, corrected distance visual acuity; UIVA, intermediate visual acuity; UNVA, uncorrected near visual acuity.

### Visual acuity

**FIGURE 1:**
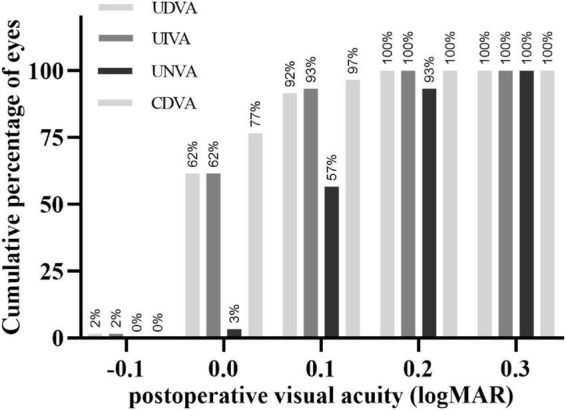
Cumulative percentage of eyes that achieved a cumulative value of monocular uncorrected distance (5 m), intermediate (80 cm), and near (40 cm) visual acuity, and corrected-distance visual acuity in logarithmic units. UDVA, uncorrected distance visual acuity; UIVA, intermediate visual acuity; UNVA, uncorrected near visual acuity; CDVA, corrected distance visual acuity.

Postoperatively, the mean logarithmic values of UDVA, UIVA, UNVA, and CDVA were 0.05 ± 0.07, 0.04 ± 0.06, 0.15 ± 0.07, and 0.03 ± 0.05, respectively (Table 1). All UDVA and UIVA values were 0.2logMAR or above; 93% (56/60) of UNVA values were 0.2logMAR or above, and all of them were 0.3logMAR or above (Figure 1). The sphere and cylinder were −0.23 ± 0.49D and −0.59 ± 0.49D, respectively.

### Contrast sensitivity

**FIGURE 2:**
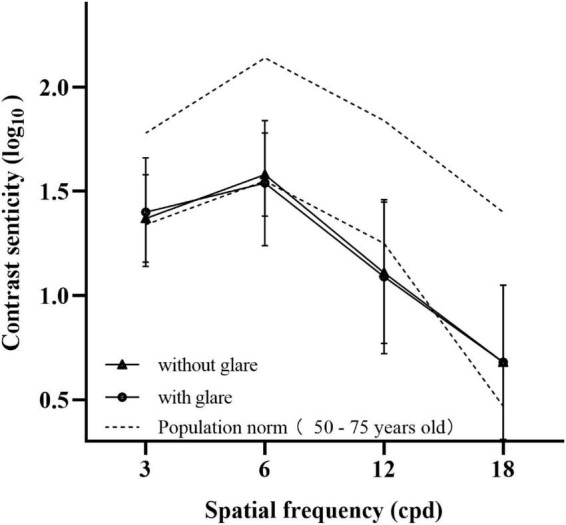
Mean value of contrast sensitivity without glare (triangle) and contrast sensitivity with glare (circle) at spatial frequencies of 3, 6, 12, and 18 cpd. The results were compared with the normal value defined previously (dotted line).

The mean logarithmic value of contrast sensitivity at spatial frequencies of 3, 6, 12, and 18 cpd without glare were 1.37 ± 0.21, 1.58 ± 0.20, 1.11 ± 0.34, and 0.68 ± 0.37, respectively. The mean logarithmic value of contrast sensitivity at spatial frequencies of 3, 6, 12, and 18 cpd with glare were 1.40 ± 0.26, 1.54 ± 0.30, 1.09 ± 0.37, and 0.68 ± 0.37, respectively. All values of the mean contrast sensitivity with and without glare were within the normal range except at a spatial frequency of 12 cpd (Figure 2) (11).

### Visual quality

**FIGURE 3:**
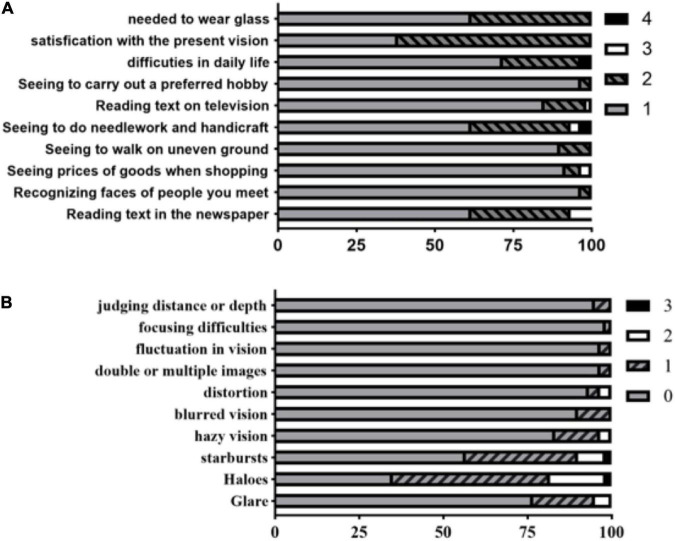
The Catquest-9SF questionnaire perceived difficulty scores in performing daily-life activities. There are four (summary scoring value) response options for the perceived difficulty levels as follows: 4 = very great difficulty; 3 = great difficulty; 2 = some difficulty; 1 = no difficulty **(A)**. The Quality of Vision (QoV) questionnaire were asked to rate 10 dysphotopsia items with 4 response levels (0, 1, 2, 3; higher score means worse photic phenomena) **(B)**.

According to the results of this subjective visual quality questionnaire survey, all the patients were very satisfied with their current vision. Approximately 65.1% (28/43) of them never wear glasses, and 34.9% (15/43) occasionally wear glasses (Figure 3A). The visual symptoms of the operative eyes, if any, were mainly glare, halos, and starbursts. However, these had no significant effect on daily life and work (Figure 3B).

The objective visual quality values of OSI, MTF cutoff, SR were 1.01 ± 0.65, 38.92 ± 10.52, 0.20 ± 0.07, respectively. All these values suggested that the patients had excellent objective visual quality after the operation.

### Higher-order aberration

The mean corneal spherical aberrations of 6-mm measurement range before and after the operation were 0.24 ± 0.10 μm (ranging from 0.07 to 0.47 μm) and 0.22 ± 0.12 μm (ranging from 0.01 to 0.51 μm), respectively, with no significant difference between them (*P* > 0.05). The mean postoperative ocular spherical aberration of 4-mm measurement range was −0.03 ± 0.07 μm, ranging from −0.23 to 0.14 μm. The mean postoperative ocular coma, trefoil, and secondary astigmatism were 0.15 ± 0.10 μm (range: 0.01–0.44 μm), 0.21 ± 0.10 μm (range: 0.05–0.50 μm), and 0.09 ± 0.07 μm (range: 0.01–0.26 μm), respectively.

### The relationship between spherical aberration and visual quality parameters

Spearman’s correlation analysis was used to evaluate the relationship between preoperative corneal spherical aberration and postoperative ocular spherical aberration with the postoperative parameters of visual quality (UDVA, UIVA, UNVA, CDVA, OSI, MTF cutoff, SR, and CS). Preoperative corneal spherical aberration was negatively correlated with postoperative UNVA and contrast sensitivity at a spatial frequency of 18 cpd under non-glare and glare (*r* = −0.403, −0.300, −0.360; all *P* < 0.05). This meant that an increase in preoperative corneal spherical aberration resulted in better postoperative UNVA (the logarithmic value of UNVA was lower), while the contrast sensitivity at 18 cpd became worse. There was no correlation between preoperative corneal spherical aberration and all the other parameters (all *P* > 0.05). Additionally, no correlation between postoperative ocular spherical aberration and any of the visual quality parameters was present (all *P* > 0.05) (Table 2).

**TABLE 2:** The relationship between preoperative corneal spherical aberration and some postoperative parameters of visual quality using Spearman’s correlation analysis.

	Preoperative corneal spherical aberration (6 mm)		Postoperative ocular spherical aberration (4 mm)	
**Parameters**	***r*** **value**	***P*** **-value**	***r*** **value**	***P*** **-value**
UDVA	0.114	0.387	-0.089	0.498
UIVA	0.035	0.791	-0.170	0.195
UNVA	-0.403	0.001*	-0.129	0.327
CDVA	0.141	0.283	-0.010	0.938
OSI	0.050	0.704	0.076	0.565
MTF cutoff	-0.088	0.505	-0.078	0.551
SR	-0.087	0.511	-0.088	0.503
3 cpd	-0.083	0.527	-0.114	0.386
6 cpd	-0.054	0.681	-0.110	0.404
12 cpd	-0.096	0.463	-0.199	0.127
18 cpd	-0.300	0.020*	-0.055	0.676
3 cpd (with glare)	-0.085	0.520	-0.072	0.584
6 cpd (with glare)	-0.146	0.266	-0.171	0.192
12 cpd (with glare)	-0.225	0.084	-0.124	0.346
18 cpd (with glare)	-0.360	0.005*	-0.162	0.216

UDVA, uncorrected distance visual acuity; UIVA, intermediate visual acuity; UNVA, uncorrected near visual acuity; CDVA, corrected distance visual acuity; OSI, objective scatter index; MTF, modulation transfer function; SR, Strehl ratio; cpd, cycle/degree; r, correlation coefficient. *P < 0.05.

### Factors affecting near vision

Spearman’s correlation analysis was used to analyze the factors affecting UNVA. The influencing factors included preoperative corneal spherical aberration, age, axial length, corneal astigmatism, postoperative sphere, cylinder, ocular coma, trefoil, and secondary astigmatism. After the spearman’s correlation analysis, factors with *P* < 0.2 were included in the further multiple linear regression, namely preoperative corneal spherical aberration (*r* = −0.403; *P* = 0.001), postoperative sphere (*r* = 0.302; *P* = 0.013), and secondary astigmatism (*r* = −0.205; *P* = 0.116). Lastly, the preoperative corneal spherical aberration was the only factor influencing UNVA after multiple linear regression analysis (Tables 3A,B).

**TABLE 3:** Spearman correlation **(A)** and multiple linear regression **(B)** were used to analyze the impact of preoperative corneal spherical aberration, age, axial length, corneal astigmatism, postoperative sphere, cylinder, ocular coma, trefoil, and secondary astigmatism on UNVA.

A. Spearman’s correlation analysis was used to analyze the factors affecting UNVA		
	***r*** **value**	***P*** **-value**
Preoperative corneal spherical aberration	–0.403	0.001**
Age	–0.091	0.491
Axial length	–0.162	0.216
Preoperative corneal astigmatism	–0.096	0.465
Postoperative sphere	0.320	0.013**
Postoperative cylinder	–0.038	0.772
Postoperative ocular coma	0.092	0.483
Postoperative ocular spherical aberration	–0.129	0.327
Postoperative ocular trefoil	0.137	0.296
Postoperative ocular secondary astigmatism	–0.205	0.116**
**B. After the spearman’s correlation analysis, factors with *P* < 0.2 were included in the further multiple linear regression**					
**Parameters**	***b***	**Standard error**	**β**	***t*** **value**	***P*** **-value**
Preoperative corneal spherical aberration	–0.223	0.087	–0.316	–2.549	0.014
Postoperative ocular secondary astigmatism	–0.131	0.125	–0.127	–1.053	0.297
Sphere	0.028	0.017	0.202	1.634	0.108

UNVA, uncorrected near visual acuity. **P < 0.2.

## Discussion

In this study, patients obtained satisfactory UDVA and UIVA values, but their UNVA was slightly insufficient, similar to previous studies (9, 10). The present study was first found that preoperative corneal spherical aberration was the only factor influencing UNVA after implanted with the TECNIS Symfony IOL, and it was negatively correlated with postoperative UNVA (logMAR visual acuity), which indicates that the greater the preoperative corneal spherical aberration, the better the UNVA. It was thought that this was caused by the larger corneal spherical aberration retaining more positive ocular spherical aberration after cataract surgery, which provided synergistic depth of focus. The depth of focus then extended to the front of the retina, compensating for the poor near vision. This result can partly explain the clinical doubts, why some patients have excellent near visual acuity, and why some have poor near visual acuity.

We also found that preoperative corneal spherical aberration was negatively correlated with contrast sensitivity at a spatial frequency of 18 cpd, indicating that the larger the corneal spherical aberration, the worse the contrast sensitivity under 18 cpd spatial frequency. This characteristic is similar to that of the visual quality of a monofocal pseudophakic eye. After implantation of negative-aberration IOLs in pseudophakic eyes, the ocular spherical aberration decreases, and the contrast sensitivity becomes better than that of no-aberration IOLs (3, 12). Therefore, we summarized that large preoperative corneal spherical aberration and residual positive ocular spherical aberration can improve near vision but impair contrast sensitivity at high spatial frequencies. This finding can guide clinicians to select appropriate IOLs based on the patient’s preoperative corneal spherical aberration and vision requirements. For patients with large preoperative plus spherical aberration, better near vision could be expected. On the contrary, if the preoperative spherical aberration is relatively small, the postoperative refraction should be targeted more myopic to achieve better near vision, or a multifocal IOL with high near-add as an alternative.

In addition, it was found in this study that preoperative corneal spherical aberration did not correlate with contrast sensitivity under 3, 6, 12 cpd spatial frequency and objective visual quality parameters, as measured by OQAS. This explains why there are no correlations between preoperative corneal spherical aberration and postoperative ocular spherical aberration with subjective visual quality parameters measured by the two questionnaires. A majority of our patients were satisfied with their visual acuity and visual quality in their daily lives. Son et al. (13) and Xu et al. (14) have found that EDOF-IOL is more tolerant to decentration and refractive errors than bifocal and monofocal IOLs. Therefore, when cataract patients want to take off their glasses after surgery and are sensitive to photic symptoms, EDOF-IOLs may be a better choice. Besides, Ruiz-Alcocer et al. (15, 16) assessed that the EDOF-IOL optical properties were more stable when a myopic ablation is introduced.

This study has some limitations. First, including both eyes from some patients may have biased the results. However, the measured parameters were analyzed individually in each eye, which mitigated this shortcoming to some extent. Second, only a type of intraocular lens was enrolled. Whether this result can be extended to other types of multifocal intraocular lenses needs further research. Lastly, although the −0.27 μm asphericity of the TECNIS Symfony IOL was designed based on a corneal spherical aberration in the range of 6 mm, it can be assumed that the measured value of ocular spherical aberration in the 4-mm range is valid because the pupils of elderly individuals become smaller over time, so the ocular spherical aberration better reflects the real-life state (17).

In summary, positive spherical aberration will benefit near-visual acuity by reducing contrast sensitivity at high spatial frequency when implanted with the TECNIS Symfony IOL.

## Data availability statement

The original contributions presented in this study are included in the article/supplementary material, further inquiries can be directed to the corresponding author.

## Ethics statement

The studies involving human participants were reviewed and approved by the Institutional Ethics Committee of Wenzhou Medical University, Zhejiang Province, China. The patients/participants provided their written informed consent to participate in this study.

## Author contributions

DW participated in the topic selection, design, data analysis, interpretation, and wrote the manuscript. CL, WG, ZL, and YiZ participated in data collection and data analysis. YuZ participated in topic selection, design, data analysis, interpretation, and manuscript revision. All authors contributed to the article and approved the submitted version.
